# Nail gun injuries to the head with minimal neurological consequences: a case series

**DOI:** 10.1186/s13256-016-0839-1

**Published:** 2016-03-16

**Authors:** Ziyad Makoshi, Fahad AlKherayf, Vasco Da Silva, Howard Lesiuk

**Affiliations:** 1 Department of Surgery, College of Medicine, Prince Sattam Bin Abdulaziz University, Al-Kharj, Saudi Arabia; 2 Division of Neurosurgery, Department of Surgery, The Ottawa Hospital – Civic Campus, Ottawa, ON Canada

**Keywords:** Brain, Nails, Neurosurgery, Penetrating trauma

## Abstract

**Background:**

An estimated 3700 individuals are seen annually in US emergency departments for nail gun-related injuries. Approximately 45 cases have been reported in the literature concerning nail gun injuries penetrating the cranium. These cases pose a challenge for the neurosurgeon because of the uniqueness of each case, the dynamics of high pressure nail gun injuries, and the surgical planning to remove the foreign body without further vascular injury or uncontrolled intracranial hemorrhage.

**Case presentation:**

Here we present four cases of penetrating nail gun injuries with variable presentations. Case 1 is of a 33-year-old white man who sustained 10 nail gunshot injuries to his head. Case 2 is of a 51-year-old white man who sustained bi-temporal nail gun injuries to his head. Cases 3 and 4 are of two white men aged 22 years and 49 years with a single nail gun injury to the head. In the context of these individual cases and a review of similar cases in the literature we present surgical approaches and considerations in the management of nail gun injuries to the cranium. Case 1 presented with cranial nerve deficits, Case 2 required intubation for low Glasgow Coma Scale, while Cases 3 and 4 were neurologically intact on presentation. Three patients underwent angiography for assessment of vascular injury and all patients underwent surgical removal of foreign objects using a vice-grip. No neurological deficits were found in these patients on follow-up.

**Conclusions:**

Nail gun injuries can present with variable clinical status; mortality and morbidity is low for surgically managed isolated nail gun-related injuries to the head. The current case series describes the surgical use of a vice-grip for a good grip of the nail head and controlled extraction, and these patients appear to have a good postoperative prognosis with minimal neurological deficits postoperatively and on follow-up.

## Background

Pneumatic nail guns are commonly used in residential construction but also easily accessible at hardware stores to public consumers. A 2005 report by the Centers for Disease Control and Prevention (CDC) reported that nail gun-related injuries have increased threefold in the USA since 1991 with an estimated 37,000 patients being seen annually in emergency departments (EDs) for nail gun-related injuries from 2001 to 2005 [8]. Males were the predominant gender treated for such injuries [8, 16] with an average age of 27 years among workers [8, 16] and 35 years among consumers [8].

The majority of traumatic injuries involved the upper extremities [8]; however, intracranial injuries have been reported as a work-related or intentional injury [1, 9]. Injuries due to penetrating trauma to the head include immediate complications via direct neurological or vascular injury, and delayed complications via vascular malformations and infection through contamination with bone or foreign body fragments.

The approach to these injuries has been largely similar to that of penetrating foreign bodies [11]. Here we present a case series of four patients presenting to our ED with penetrating nail gun injuries to the head with minimal neurological complications as well as approach considerations and surgical technique in removing these foreign bodies.

## Case presentation

### Method

Preoperatively, all patients received antibiotics administered intravenously and a tetanus shot. After imaging and evaluation for vascular injury, the patients were taken to the operating room (OR). Intraoperatively, the patients were positioned for proper exposure; we shaved the head of each patient and prepped around the injury site(s), each patient’s head was again examined to identify the entry point of all the foreign bodies.

A skin incision was made over the nail entry point and extended down to the nail. After dissection down to the cranial vault the skull was again examined to identify the entry point of all foreign objects. After debridement, a small craniectomy was done around the nail followed by opening of the dura around the nail in a cruciate fashion. The nail was removed carefully to avoid excessive movement of those nails and injury to surrounding structures. When the nail was difficult to grasp or was met with resistance, a vice-grip was used to grip the head of the nail for extraction.

After removing the nail(s), some sites had minimal bleeding from minor cortical vessels which was easily controlled with bipolar cautery. The entry point was inspected for several minutes and irrigated with normal saline followed by hydrogen peroxide or antimicrobial solution. Each patient’s head was positioned for optimal approach for each injury; after extraction at one site each patient’s head was again positioned for an optimal approach to other sites in the cases with multiple injuries and we proceeded in a similar fashion.

After extraction of all foreign objects, the nails were examined to ensure they were intact and that the count was complete and no further objects were left in the cranial vault. In the first case of ten nails, an intraoperative skull X-ray was used to evaluate the removal of all foreign objects. After establishing complete removal and control of bleeding, the wound was closed in the typical fashion. All patients received a 1-week postoperative course of phenytoin as per our institutional practice; however, no patient received prophylactic antiepileptic medication prior to surgery or beyond 1 week after surgery as there was no clinical evidence of seizure activity in any of our patients. All patients underwent a postoperative computed tomography (CT) scan to evaluate for bleeding and complete removal of all foreign objects, and the nails were sent for microbiology testing.

### Case 1

**Fig. 1:**
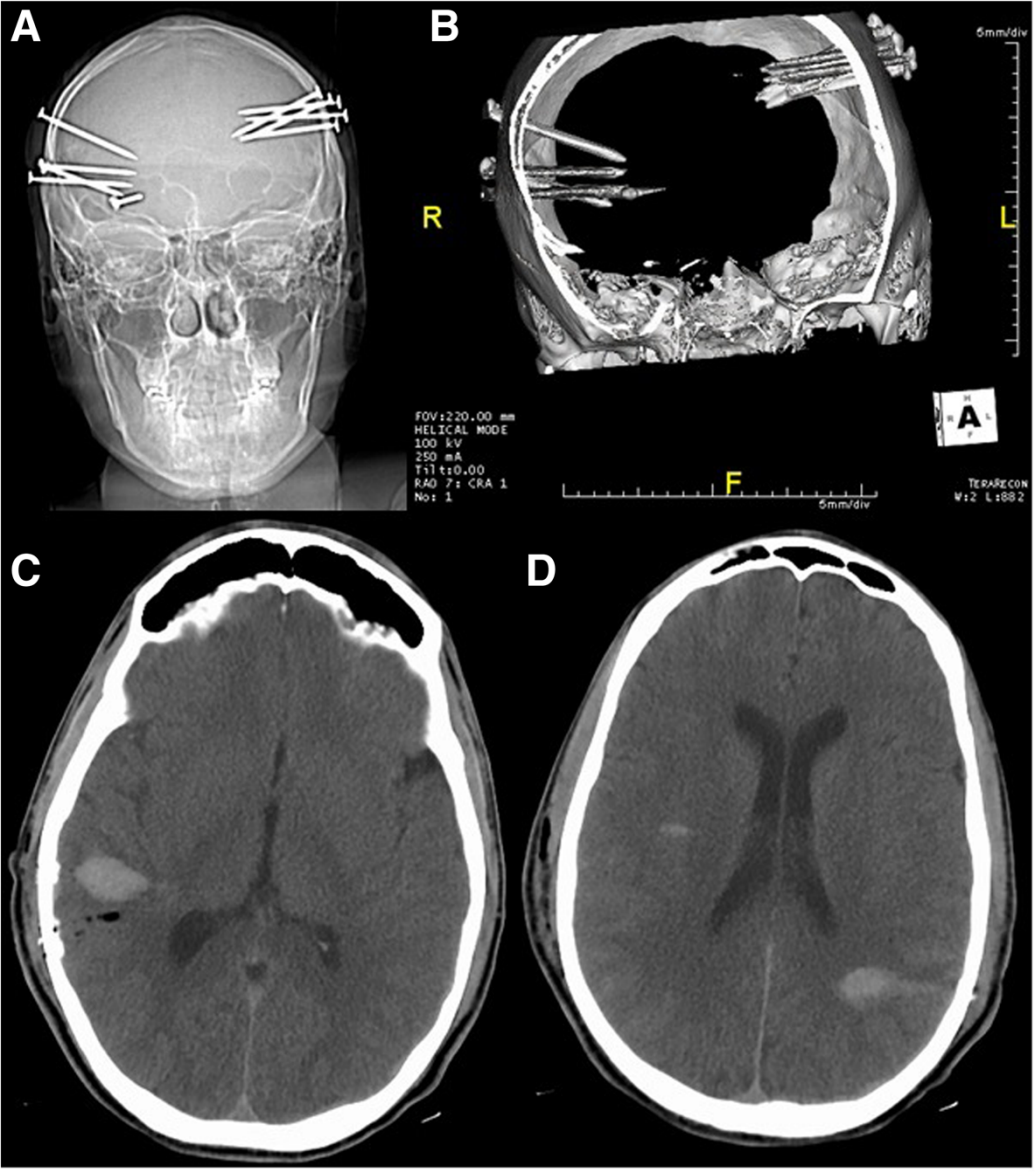
**a** Computed tomography scout. **b** Computed tomography three-dimensional reconstruction. There are ten nails projected over the temporo-parieto-occipital area bilaterally, five on each side, these caused significant artifacts on computed tomography and computed tomography angiogram (not shown here), but no major vessel injury was identified and there was evidence of parietal subarachnoid bleed. **c, d** Postoperative axial computed tomography. Multiple foci of intraparenchymal hemorrhage and associated subarachnoid hemorrhage seen in the left parietal and right frontal-temporal-parietal regions. Multiple tiny calcified bodies were now noted over the left parietal region; they probably represented small bone fragments introduced at the time of penetrating injury. These were obscured on the prior study due to metallic artifact

**Fig. 2:**
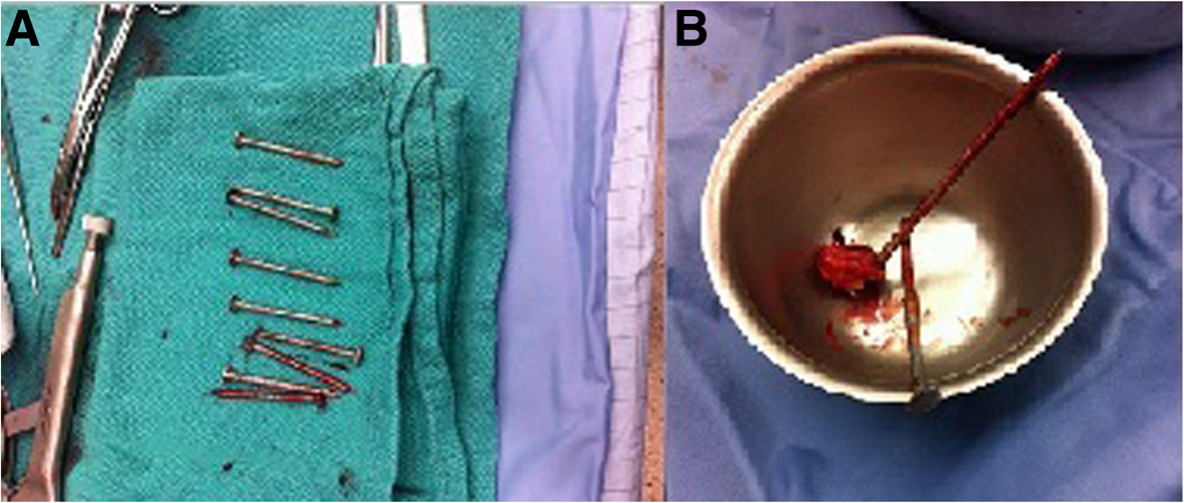
**a** Case 1. Nine out of the ten nails extracted from the patient; the remaining nail was sent for microbiology testing. **b** Case 2. Three-inch (7.62 cm) nails extracted after craniectomy

A 33-year-old, right-handed white man with a history of severe depression was transferred to our Neurosurgical unit after he shot himself in the head with a nail gun ten times. He was alert on arrival and described that he shot himself on the right side five times in different locations, and then shot himself on the left side in another five locations. Examination revealed left CN VI, VIII, XI and XII injury. A motor examination showed that he had slight weakness on his left side (4/5). A head examination revealed only eight identifiable nails at the surface of his skull. He underwent a CT and CT angiogram (Fig. 1) which showed ten nails in his temporo-parieto-occipital area bilaterally, five on each side, with no evidence of major vessel injury and he was subsequently taken for surgical extraction of the nails (Fig. 2a).

### Case 2

**Fig. 3:**
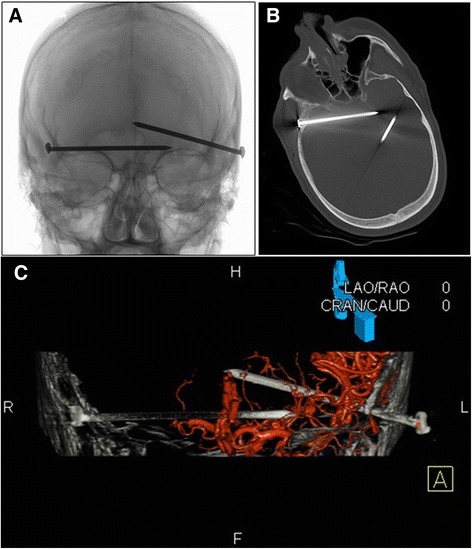
**a** Skull X-ray. **b** Computed tomography head – bone window showing two nails penetrating the calvarium on each side. There was evidence of subarachnoid hemorrhage on computed tomography. **c** Cerebral angiogram three-dimensional reconstruction. Three-dimensional rotations re-demonstrated the presence of bilateral temporal horizontal metallic nails coursing adjacent to the main proximal intracranial vessels. No definite contrast extravasation, arterial occlusion, stenosis or pseudoaneurysm was identified

A 51-year-old white man with known alcohol abuse presented to our ED with self-inflicted bilateral temporal injury using a 3-inch (7.62 cm) nail gun (Fig. 2b) after being involved in what seemed to be a social dispute with his partner. On arrival in our ED, his Glasgow Coma Scale (GCS) score was 8/15 but it soon decreased to 6 and he was intubated. Before surgical exploration, he underwent a CT, CT angiogram and cerebral angiogram (Fig. 3), which showed two nails penetrating his calvarium on each side and no definite contrast extravasation or direct vascular injury.

### Case 3

**Fig. 4:**
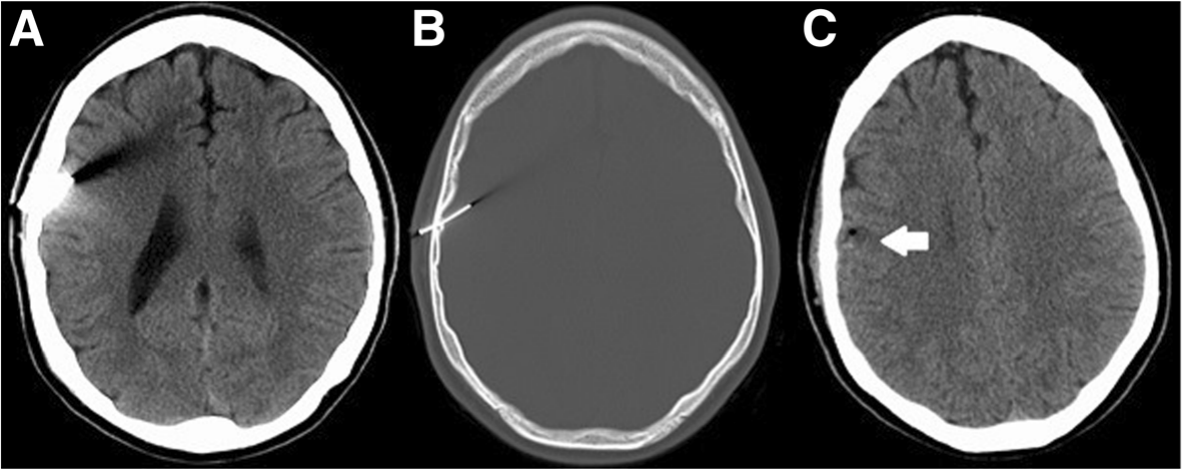
Computed tomography of the patient’s head without contrast. **a** Brain window. **b** Bone window. A metallic nail in the right parietal bone and parietal lobe in the preoperative study which is removed in the postoperative study (**c**) with small air pocket and extra-axial hematoma in the same level

A 22-year-old, white right-handed man, with a known history of schizophrenia and previous history of psychotic episodes, had apparently attempted to kill himself by placing a nail gun to his head and pulling the trigger. Fortunately, this was a Brad Nail Gun ejecting approximately 1.5-inch (3.81 cm) long nails of a small diameter, so although the nail did penetrate his skull and pierce his right frontal brain parenchyma, there was no significant bleeding associated with it, nor was there a significant clinical deficit. He did not lose consciousness and on presentation to our ED he was neurologically intact. He underwent CT of his head without contrast (Fig. 4) and was taken to our OR.

### Case 4

**Fig. 5:**
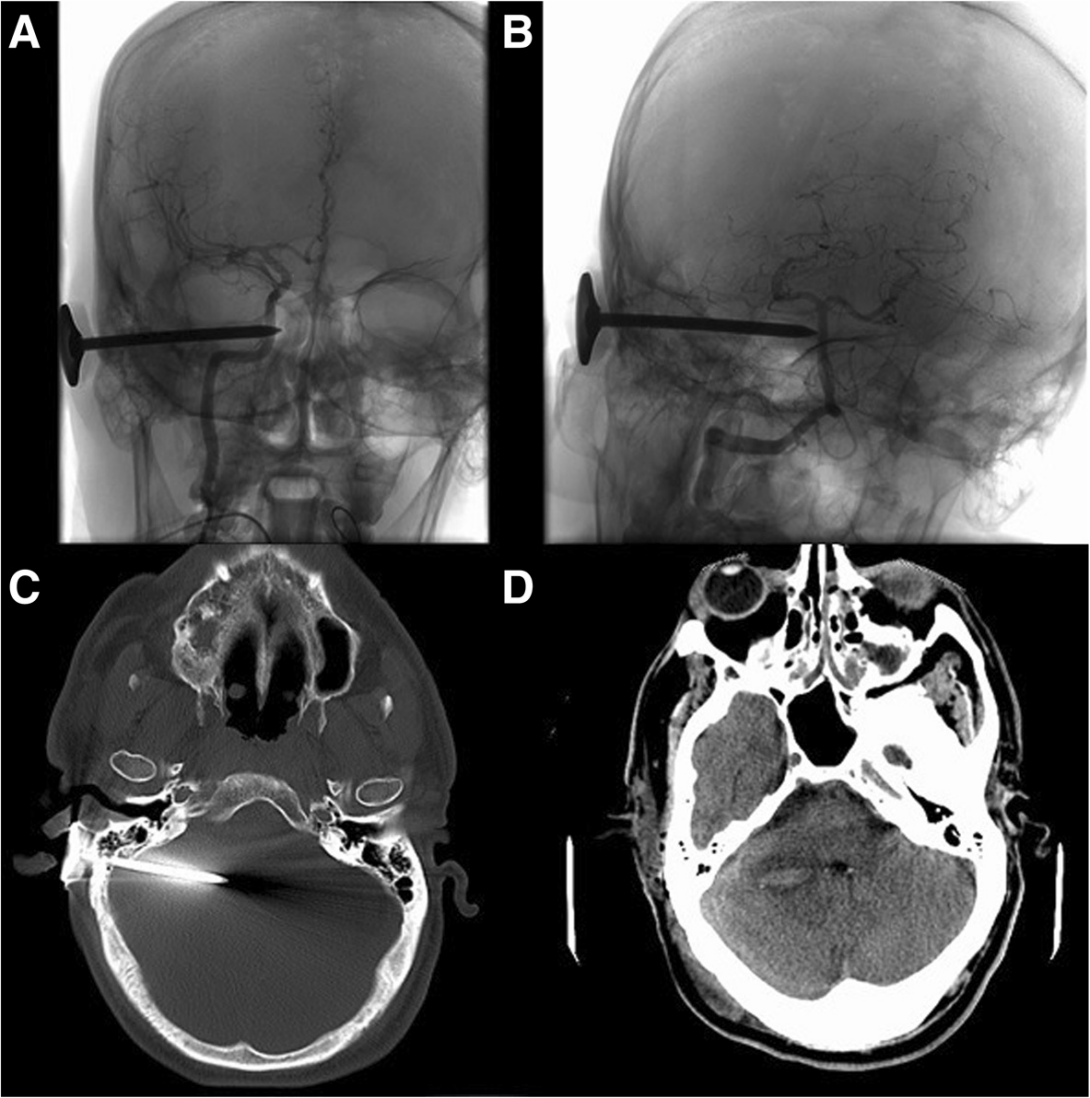
Cerebral angiogram. **a** Right internal cerebral artery. **b** Right vertebral artery. The metallic foreign body transects the superior aspect of the right transverse sinus as it merges into the right sigmoid sinus. There is no active extravasation of contrast or evidence of major arterial compromise. **c** Preoperative computed tomography of the patient’s head (bone window). **d** Postoperative computed tomography of his head. Small amount of hemorrhage noted along the track of the removed foreign body from the right posterior fossa and evidence of a small intraventricular hemorrhage

A 49-year-old white man sustained a self-inflicted nail gun injury that entered the intracranial cavity at the posterior aspect of his right ear lobe. The nail produced an opening in his earlobe and it was inserted up to the nail head inside his cranial cavity. It came with a metallic collar measuring 1.5 cm in diameter that pushed the skin of his scalp against the bone. Due to a low GCS the patient was intubated. When he was off sedation, an examination showed no neurological deficits. A CT and cerebral angiogram (Fig. 5) were obtained which showed the foreign body had transected the superior aspect of his right transverse sinus; however, there was no active extravasation of contrast or evidence of major arterial or venous compromise and he was taken to our OR.

## Results

Postoperatively these patients recovered without any major complications. Case 1 had no neurological deficits postoperatively; neuropsychological assessment showed moderate-to-severe cognitive impairment with improvement upon assessment at 3 months with persistent moderate cognitive impairment and a score of 26/30 on the Montreal Cognitive Assessment (MoCA) test. Case 2 experienced a few days of confusion and aggressive behavior that resolved; postoperative neuropsychological assessment showed difficulties with complex attention, memory and executive functions, these improved at reassessment at 5 months with persistent mild-to-moderate cognitive impairment and a MoCA score of 20 + 1/30 with the belief that some of these deficits were probably premorbid. Case 3 had no neurological deficits postoperatively or on follow-up at 5 months but continues to be followed by psychiatry for issues regarding his chronic schizophrenia. Case 4 had some unsteadiness of gait during the postoperative period that resolved; he received psychological assessment at another institute for which the records could not be obtained. All patients received physiotherapy as part of their rehabilitation. Follow-up imaging of these patients at 3 to 5 months with a CT of their head without contrast showed no hemorrhage and areas of encephalomalacia corresponding to the injury sites, as expected.

## Discussion

Nail gun injuries are commonly reported in the workplace [18]. Trigger mechanisms include contact triggers whereby a nail is discharged when there is depression of the nose piece of the gun and the trigger simultaneously; this type of mechanism has been associated with increased risk of accidental injury to self and others [19]. However, a sequential actuation system has been shown to decrease the incidence of non-intended firing whereby the nail is only discharged if the nose element is depressed before pressing the trigger. The air pressure on these devices can be between 80 and 120 psi, often sufficient enough to penetrate the scalp [15], and can reach projectile speeds of up to 100 to 150 m/second [17]. Experiments with high pressure injuries on human hand cadavers showed that the lateral distribution of energy is limited and that the majority of the trauma occurs along the path of penetration [15].

The aforementioned cases of nail gun injuries, however, were all self-inflicted and in the setting of psychiatric disorder or substance abuse. Initial impact during such injury is usually painless [15]. The patients often present fully conscious; our patients’ history was informative in assessing the true number of injuries confirmed on subsequent imaging. No signs of active hemorrhage were present, and the patient’s status in all four cases was stable to warrant complete workup including imaging to identify foreign objects and angiogram to assess for vascular injury. Mortality following these injuries is low, and those reported in the literature are due to concomitant injury to other parts of the body [7] or penetration with a larger object such as a steel rod [4].

The most common complications reported after penetrating nail gun trauma are vascular injury [2, 10, 13, 20] and intracranial hemorrhage [6, 21, 23]. When injury to a major intracranial artery has occurred these can lead to substantial subarachnoid and intraventricular hemorrhage with subsequent complications including hydrocephalus, ischemia and death [21]. A favorable outcome has been reported in the case of basilar artery injury when a clot acted as a tamponade for the vascular injury and care was taken, intraoperatively, to obtain distal control using vascular clipping and preparations for proximal control using balloon occlusion in the event extravasation occurred during extraction of the nail [10]. Reports of venous injuries show variable outcomes; a case involving injury to the superior sagittal sinus with successful nail extraction and venous repair had a good outcome with no neurological deficits [22], while a nail gun injury involving the left transverse sinus resulted in residual right hemiparesis even after extraction of the nail and repair of the venous injury [2].

Development of cerebral aneurysms and pseudoaneurysms have been reported [5, 14, 21, 24]; these may be obvious on postoperative imaging [24] or may develop in a delayed fashion [5]. Involvement of a cerebrovascular neurosurgeon and interventional neuroradiologist is indicated in these cases and management should be individualized based on location, size, and characterization, and may include observation, surgical clipping, or endovascular coiling as indicated.

An approach to patients with nail gun injuries involves detailed history and neurological examination, and examination for other injuries. When patient stability permits, imaging with CT and angiogram should be performed to assess for number, location, and associated vascular injury and for surgical planning. Although injuries from nail guns are often considered “clean” with report of successful removal without antibiotic prophylaxis [23], we recommend tetanus prophylaxis and antibiotics in these patients in light of the high rate of infection reported in other penetrating brain injuries [3, 12] and possible introduction of skin, bone or foreign body fragments into the wound [11]. The surgical approach to these injuries should include proper exposure through craniotomy for direct visual inspection of injury sites. Care should be taken when removing these objects to avoid additional injury with extraction, and the injury site should be properly examined to assess for hemorrhage after removal with thorough irrigation and debridement of the wound site. A vice-grip in our experience has been helpful in getting control of the nail head for a smooth removal even after exposing the nail intraoperatively to limit injury to surrounding structures during nail extraction. An intraoperative X-ray may be indicated when multiple sites are present to evaluate for any remaining foreign objects.

## Conclusions

Nail gun injuries are often self-inflicted and associated with a background of a psychiatric disorder. Involvement of the appropriate services during hospital care, including psychiatric evaluation, is indicated in these settings. Proper evaluation with history and physical examination, as well as vascular imaging assists in management planning for these patients and should be obtained upon presentation when possible. Craniotomies and wound debridement is the standard approach for penetrating injuries and the use of vice-grips for removal of nails in our experience facilitates nail extractions with good control. Although presentation is variable among patients, most present with minimal neurological deficits, mortality and morbidity is low for surgically managed isolated nail gun-related injuries to the head and these patients appear to have a good postoperative prognosis with proper management.

## Consent

Written informed consent was obtained from the patients for publication of this case series and accompanying images. A copy of the written consent is available for review by the Editor-in-Chief of this journal.
